# A demographic scaling model for estimating the total number of COVID-19 infections

**DOI:** 10.1093/ije/dyaa198

**Published:** 2020-12-08

**Authors:** Christina Bohk-Ewald, Christian Dudel, Mikko Myrskylä

**Affiliations:** 1 Max Planck Institute for Demographic Research, Rostock, Germany; 2 Center for Social Data Science, University of Helsinki, Helsinki, Finland

**Keywords:** COVID-19, infection, prevalence, bias assessment, local seroprevalence studies, indirect estimation

## Abstract

**Background:**

Understanding how widely COVID-19 has spread is critical information for monitoring the pandemic. The actual number of infections potentially exceeds the number of confirmed cases.

**Development:**

We develop a demographic scaling model to estimate COVID-19 infections, based on minimal data requirements: COVID-19-related deaths, infection fatality rates (IFRs), and life tables. As many countries lack IFR estimates, we scale them from a reference country based on remaining lifetime to better match the context in a target population with respect to age structure, health conditions and medical services. We introduce formulas to account for bias in input data and provide a heuristic to assess whether local seroprevalence estimates are representative for the total population.

**Application:**

Across 10 countries with most reported COVID-19 deaths as of 23 July 2020, the number of infections is estimated to be three [95% prediction interval: 2–8] times the number of confirmed cases. Cross-country variation is high. The estimated number of infections is 5.3 million for the USA, 1.8 million for the UK, 1.4 million for France, and 0.4 million for Peru, or more than one, six, seven and more than one times the number of confirmed cases, respectively. Our central prevalence estimates for entire countries are markedly lower than most others based on local seroprevalence studies.

**Conclusions:**

The national infection estimates indicate that the pandemic is far more widespread than the numbers of confirmed cases suggest. Some local seroprevalence estimates largely deviate from their corresponding national mean and are unlikely to be representative for the total population.

**Key Messages**

- The demographic scaling model facilitates the timely monitoring of the spread of the COVID-19 pandemic in many settings.
- The demographic scaling model allows estimation of the total number and prevalence of COVID-19 infections on the country level with and without accounting for bias in input data on deaths and infection fatality rates.
- The demographic scaling model is broadly applicable in contexts with both rich and poor data through minimal input data requirements, which make it a complement or an alternative to more complex methods.
- The estimates for the 10 countries with most reported COVID-19 deaths as of 23 July 2020 are uncertain and vary across countries, but consistently indicate that the pandemic is more broadly spread than the numbers of confirmed cases suggest.
- The demographic scaling model can also be used to indicate whether local seroprevalence measurements could be representative of the total population by assessing how much bias would be required in its input data on deaths and infection fatality rates in order to reproduce them.

## Introduction

The number of COVID-19 infections is a key indicator for understanding the spread of the pandemic. Although this indicator is potentially higher than the number of confirmed cases, it is largely unknown. Existing seroprevalence studies for COVID-19 have largely relied on non-representative samples,^1–4^ and population-representative studies are only slowly becoming available.^5^ Other approaches to estimate the spread of COVID-19 rely on complex statistical methods^6^^,^^7^ that typically have high data demands.

We introduce a demographic scaling model to nowcast the number of COVID-19 infections in a population on the country level. It is inspired by indirect estimation techniques^8^ and methods to model and forecast mortality^9–11^ from the field of demography. Our approach serves two major aims. It is designed first to estimate the total number and prevalence of COVID-19 infections, and second to assess whether local seroprevalence measurements could be representative of the total population. Depending on the data available, our model can be regarded either as complement or as an alternative to the more complex models that already exist for estimating the numbers of infections.

Our model can be broadly applied in contexts with both rich and poor data, as it requires minimal input: the number of COVID-19-related deaths for the population of interest; age-specific infection fatality rates (IFR; deaths over infections) from a reference population; and life tables used to scale IFRs to match the target population with respect to age structures, health conditions and medical services. Borrowing and scaling IFRs is necessary, as IFRs are not available for many countries.

We apply the demographic scaling model to estimate the total number and prevalence of COVID-19 infections in 10 countries that have the most reported COVID-19 deaths as of 23 July 2020. We also compare local seroprevalence for Germany, the USA and Italy with our nationwide infection prevalence estimates. Given the rapid progress of the COVID-19 pandemic, we refer the reader to latest results and the R source code to (re)produce them at [https://github.com/christina-bohk-ewald/demographic-scaling-model].

This research project does not require ethics approval as it uses only macro data that are freely available online.

## Methods

### The demographic scaling model

The demographic scaling model estimates the total number I and prevalence λ of infections. We start with the basic identity that represents the age-specific number of infected:

(1) $$I_{x}=P_{x}\;\cdot \;\lambda_{x},$$

where I is the unknown number of infected, P is the known population size, λ is the unknown fraction of the infected population, and x denotes the age group. We estimate $\lambda_{x}$ by using the equation: $D_{x}={\mathrm{IFR}}_{x}\cdot \;P_{x}\;\cdot \;\lambda_{x}$, where D is the number of deaths and IFR is the infection fatality rate. We rearrange the equation to get $\lambda_{x}=D_{x}/[{\mathrm{IFR}}_{x}\cdot \;P_{x}]$, and estimate the total number of infected by:

(2) $$I=\sum_{x}P_{x}\cdot \lambda_{x}$$

Replacing $\lambda_{x}$ with its definition yields:

(3) $$I=\sum_{x}D_{x}/{\mathrm{IFR}}_{x}$$

The model estimates infections at a discrete time point, using input data that most closely refer to that time point. If data on time-varying IFRs becomes available, this can be incorporated in the model.

The key challenge is to arrive at credible estimates of ${\mathrm{IFR}}_{x}$ and $D_{x}$ , as the demographic scaling model assumes that COVID-19 deaths and IFRs are fairly accurately recorded and that IFRs borrowed from a reference country reflect the true IFRs of the target country after appropriate scaling. We show below how our method can be used if these assumptions are violated.

### Credible estimates of IFR_x_ and D_x_

${\mathrm{IFR}}_{x}$ are not available for many countries. To obtain country-specific estimates of ${\mathrm{IFR}}_{x}$ we borrow them from a reference country and scale them to better match the context in a target population with respect to age structures, underlying health conditions and medical services. This scaling is particularly important as the presence of older age and underlying health conditions—such as cardiovascular diseases, diabetes, chronic respiratory diseases, hypertension and neoplasms—increase the risk of death with a given COVID-19 infection.^12^^,^^13^ In addition, the ability of health care systems to treat illnesses effectively may also affect COVID-19 mortality and vary among countries.

To account for the differential vulnerability to COVID-19 in each target population, we map IFRs between a reference and a target population based on their remaining lifetime, denoted by $e_{x}$. Remaining lifetime is a function of chronological age, health conditions and a health care system’s effectiveness in curing diseases.^14^ The younger people are, the fewer underlying health conditions they have; and the more effective medical care is in treating illnesses, the more life-years people have left.

We assign the same infection fatality rate (IFR) to people of two countries who have, on average, the same number of life-years left ($e_{x}$):

(4) $$IFR_{e_{x}}^{\mathrm{COI}}=IFR_{e_{x}}^{RC},$$

where COI denotes the country of interest and RC denotes the reference country. For example, if 70-year-olds in a reference country have, on average, the same number of life-years left as 75-year-olds in a country of interest, the infection fatality rate of the 70-year-olds in the reference country is used for the 75-year-olds in the country of interest.

We assume that remaining lifetime is a good proxy for overall health conditions and medical services in a population, and note that it is readily available for many countries. Scaling IFRs should be the more effective, the more similar the overall structure and distribution of causes of death are in a target and reference population, even more so when these diseases affect the vulnerability to COVID-19.^12^^,^^13^ The scaling should not work well when one of the two countries has disproportionately many people dying from, for example, external causes (suicide, homicide and accidents) which have not been shown to be related to COVID-19, but which have a big effect on remaining lifetime. In most countries, however, mortality of people at ages above 50—being most vulnerable to COVID-19 and, consequently, particularly relevant for the proposed method—is mostly driven by cardiovascular diseases, neoplasms, and chronic respiratory diseases.^15^ Therefore we consider it reasonable to assume that scaling IFRs should be effective to approximate the true IFRs in many countries of interest.

Whereas COVID-19 deaths are available in total numbers for many countries worldwide,^16^ they are often not available by age. We disaggregate total deaths into age groups using a global average pattern over age that we derived from data provided by Dudel *et al*.^17^ (Supplementary Appendix 3, available as Supplementary data at *IJE* online).

### Accounting for bias in input data

Our input parameters on COVID-19 deaths and IFRs are both prone to bias. The number of COVID-19 deaths may be under- or overestimated. Reporting delays and inconsistent practices for defining and testing COVID-19 deaths are among the key sources of error. (Scaled) IFRs may also be under- or overestimated. In addition to reporting, classification and testing errors, the population structure by age and precondition, the performance and occupancy rate of medical services and the taken control measures and their acceptance in a population, are main factors that impact on both the IFRs in the reference population and their scalability to other countries of interest.

If this bias could be quantified for (i) deaths, for example through emerging studies on COVID-19-related excess mortality,^18–20^ and for (ii) IFRs, for example through more, better and consistent surveillance data becoming available,^21^ the demographic scaling model could account for it in formula (3) by introducing the relative amount of under- or overestimation B. For example, if B denotes the relative amount of misreporting deaths, our estimate of the number of infections with bias $I^{B}$ can be written as:

(5) $$I^{B}=B\sum_{x}D_{x}/{\mathrm{IFR}}_{x}=B\cdot I^{T},$$

assuming that bias affects all ages to the same extent and that $I^{T}$ is the true number of infections. Equation (5) shows that a biased estimate of infections could be adjusted in order to derive the true number of infections, $I^{T}=I^{B}/B$. For example, if B is below or above one, then COVID-19 deaths are under- or overreported, respectively. Bias in IFRs can be handled in a similar fashion. Note that the potential bias in our infection estimates could be unequally distributed by age due to, for example, differential vulnerability and testing coverage by age.^22^

### Assessing whether local seroprevalence estimates are representative of total population

Adapting equation (5) allows us to assess whether local seroprevalence estimates $I^{S}$ could be representative of the total population, by quantifying the bias B that would be required in deaths and IFRs in order to match our estimates, $I^{E}$. We can express our estimates $I^{E}$ in terms of the local seroprevalence estimates $I^{S}$ and an under/overestimation factor B as $I^{E}=B\sum_{x}D_{x}/{\mathrm{IFR}}_{x}=B\cdot I^{S}$. Then B is simply given by $I^{E}$/$I^{S}$. The two estimates $I^{E}$ and $I^{S}$ might be considered inconsistent if B is very high or low. This inconsistency can indicate that the local seroprevalence estimate $I^{S}$ was not representative of the total population, or that the COVID-19 deaths (or IFRs) used in our estimate for $I^{E}$ are biased by a factor of B (or 1/B). For instance, reproducing an $I^{S}$ that is much larger than our original $I^{E}$ could be achieved by assuming that COVID-19 deaths are underestimated by a factor of B. If this estimate of underreporting COVID-19 deaths is large, the interpretation that $I^{S}$ is not representative of the total population may be more plausible. What is ‘large’ is likely to be context dependent, and information on estimates of excess mortality^18–20^ may be useful when making this judgment.

### Empirical data for estimating COVID-19 infections

We use the demographic scaling approach (i) to estimate COVID-19 infections for the 10 countries that have reported most COVID-19 deaths as of 23 July 2020, and (ii) to assess whether recent local seroprevalence studies for the USA, Italy and Germany are likely to be representative of the corresponding total population. As input data, we use (i) 2019 population counts and life tables of the United Nations (UN),^23^ (ii) accumulated COVID-19 deaths from Johns Hopkins University Center for Systems Science and Engineering (CSSE),^16^ and (iii) IFRs for Hubei, China.^21^

The UN^23^ provides remaining lifetime of China, which is comparable to remaining lifetime of Hubei.^24^ We select Hubei’s IFRs because they were the first to be published in a peer-reviewed journal, are based on a relatively large population sample, account for potential biases, and have passed several robustness checks.^21^ For example, Verity *et al*.^21^ account for potential bias in IFRs caused by, for example, different surveillance settings. We acknowledge, however, that there may be problems with Hubei’s IFRs and note that our method can flexibly use better IFR estimates when they may emerge over time. We use the lower and upper bound of the 95% credible interval of Hubei’s IFRs, which have been derived through Bayesian analysis, to generate the boundaries of the 95% prediction interval of our infection estimates. The uncertainty estimates of our model might be too large (or conservative) as we do not account for covariance in IFR across ages. As the IFRs are provided by 10-year age groups, 0 - 9, 10 - 19, …, 80+, we prepare all input data to match them. Model details and additional findings are given in Supplementary Appendices 1-7, available as Supplementary data at *IJE* online.

## Results

**Figure 1:**
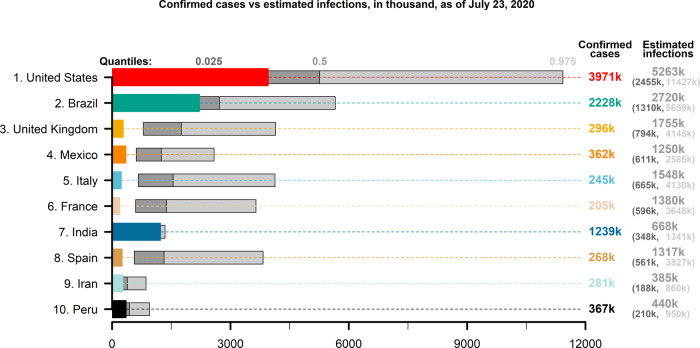
Confirmed cases versus estimated infections. Confirmed cases (non-floating, coloured bars) and estimated COVID-19 infections (quantiles 0.025, 0.5 and 0.975; floating, grey bars) for the 10 countries that have the largest numbers of reported deaths from COVID-19 as of 23 July 2020. Own calculations using data from Verity *et al*.,^21^ United Nations World Population Prospects^23^ and Johns Hopkins University Center for Systems Science and Engineering^16^

Figure 1 shows the infection estimates for 10 countries that have reported most COVID-19 deaths as of 23 July 2020: the USA, Brazil, the UK, Mexico, Italy, France, India, Spain, Iran and Peru. The infection estimates are uncertain and vary across countries, but consistently indicate that the pandemic is more broadly spread than the numbers of confirmed cases suggest. Across the 10 countries in our sample, the total number of infections is estimated to be approximately three [95% prediction interval: 2–8] times higher than the number of confirmed cases. For example, for the USA, which has 4 million confirmed cases, we estimate that the total number of infections might range from approximately 2.5 million to 11.4 million, with a central estimate of 5.3 million infections, which is only slightly more than the number of confirmed cases. For a large number of countries, the central infection estimate is more than three times higher than the number of confirmed cases. For example, for France, we estimate that there are approximately 1.4 million infections [95% prediction interval: 0.6–3.7 million], whereas the total number of confirmed cases, 205 000, is almost one seventh of the estimated infections. India, where the pandemic struck relatively late and testing has been comparatively limited, stands out in this context, as our central estimate of infections, 668 000 [95% prediction interval: 348 000–1.3 million], is smaller than the number of confirmed cases, 1.2 million. Case fatality rate in India is low, lower than in well-performing South Korea and in many other high-income countries.^25^ This suggests that it is possible that a comparatively large number of COVID-19 deaths may be undetected in India, which would bias our estimates downward. Moreover, the time from disease onset to death from COVID-19, which can be several weeks,^26–28^ and the rapidly increasing number of confirmed cases in India, could also partially explain why our estimates are low compared with the confirmed cases. Supplementary Appendix 7, available as Supplementary data at *IJE* online, provides information about test coverage, which appears to increase with the duration of the pandemic and the number of confirmed cases.

**Figure 2:**
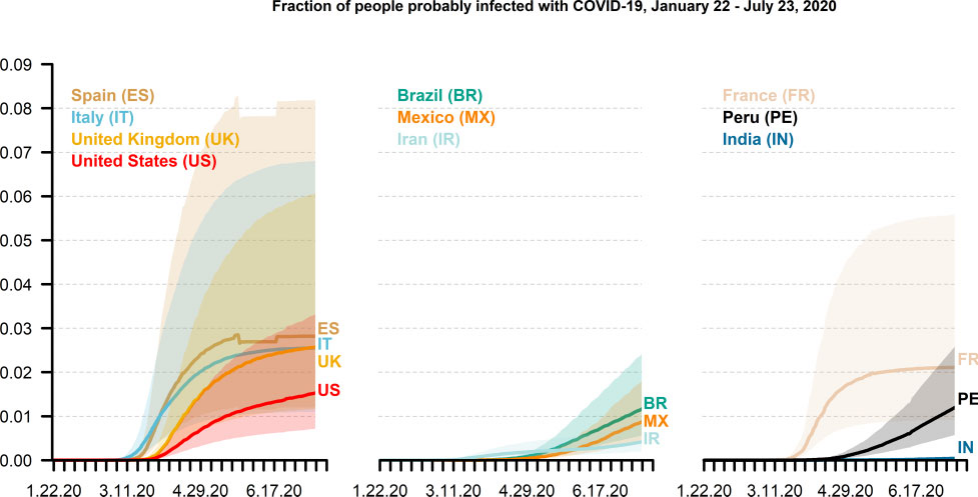
Estimated COVID-19 infection prevalence. Estimated population share of COVID-19 infections (quantiles 0.025, 0.5 and 0.975), from 22 January to 23 July 2020, for the 10 countries that have the largest numbers of reported deaths from COVID-19 as of 23 July 2020. Own calculations using estimates of Verity *et al*.,^21^ United Nations World Population Prospects^23^ and Johns Hopkins University Center for Systems Science and Engineering^16^

Figure 2 shows that the COVID-19 prevalence is estimated to have increased for most countries in our sample over time. As of 23 July 2020, we find the central prevalence estimate to be on average 1.6% [95% prediction interval: 0.7–3.9%]. It ranges: from 2.8% in Spain; to between 2% and 2.6% in the UK, Italy and France; to approximately 1% and 1.6% in the USA, Peru, Brazil and Mexico; and to 0.5% or less in Iran and India. The upper bound includes values as high as 8.2%, 6.8% and 6.1% for Spain, Italy and the UK, respectively. It is striking that infection prevalence appears to stabilise for Spain, France and Italy, whereas it continues to increase for the other countries in our sample.

The relatively wide prediction intervals for the estimated number and prevalence of COVID-19 infections reflect the high level of uncertainty in input data on COVID-19 during the early stages of the pandemic. Despite the high uncertainty, the bounds provide useful information. In most cases the lower bound is well above the number of confirmed cases, suggesting that the latter underestimates the number of infections. The upper bound, high as it often is, is also important information that should be factored in as a possibility in planning. Moreover, for estimates of change over time, the uncertainty may be lower than for a single point in time if the sources of error stay constant.

As the COVID-19 prevalence is much higher according to local seroprevalence studies for the USA, Italy and Germany than our model estimates, we assess their representativeness with our model by showing how many more COVID-19 deaths would have been required to match them. For example, Bendavid *et al*.^3^ reported in the middle of April 2020 a seroprevalence between 1.1% and 5.7% for Santa Clara County in the USA, compared with our central estimate of 0.4% for the entire USA at the same time (17 April 2020). Assuming the seroprevalence estimate of 1.1% is correct, only one in three COVID-19-related deaths would have to be registered. Bendavid *et al*.^3^ cite a seroprevalence of 10% for the city of Robbio in Italy, and a seroprevalence of 14% for the German municipality of Gangelt. Table 1 shows that to be compatible with our central prevalence estimates of 1.7% in Italy and 0.2% in Germany, only one in five and fewer than two in 100 COVID-19-related deaths would have to be recorded in Italy and Germany, respectively. However, when using the upper bound of our prevalence estimates, only one in two COVID-19-related deaths would have to be missed for the USA and Italy, which is possible; whereas for Germany, the number is still unrealistically high. Either way, the infection estimates based on local seroprevalence studies appear to be much higher than our prevalence estimates, which may indicate that they are not representative of the total population.

**Table 1:** Bias required in reported COVID-19 deaths to reproduce local seroprevalence estimates

Country	Seroprevalence estimate	Scaling model estimate	**B (bias required)**
USA (Santa Clara)	3%	0.4%	0.15
Italy (Robbio)	10%	1.7%	0.20
Germany (Gangelt)	14%	0.2%	0.01

Central COVID-19 prevalence estimates for middle of April 2020, percent, according to local seroprevalence studies and our demographic scaling model, for the USA, Italy and Germany. Also shown is the amount of bias (B; here: under-reporting) that would be required to explain their discrepancy. For example, a bias of 0.2 for Italy could suggest that only one in five COVID-19 deaths would have been reported in order to explain the seroprevalence estimate with our scaling model estimate. Data source for seroprevalence estimates: Bendavid *et al*.^3^ Own calculations using estimates of Bendavid *et al*.,^3^ Verity *et al*.,^21^ United Nations World Population Prospects^23^ and Johns Hopkins University Center for Systems Science and Engineering as of 17 April 2020.^16^

## Discussion

The actual number of infections is among the key unknowns of the COVID-19 pandemic. Several studies have provided infection estimates, based either on local seroprevalence measurement^3^^,^^4^ or complex statistical models.^6^^,^^7^ None of the identified studies have provided a broadly applicable data-based approach that estimates COVID-19 infections using only a few inputs, and that takes into account cross-country differences in age structures, health conditions and health care systems.

We have developed a demographic scaling model to estimate COVID-19 infections on the country level, based on modest data requirements, allowing its application also in contexts with poor data. Our model estimates vary across the 10 countries with most COVID-19 deaths as of 23 July 2020, but consistently point in the same direction, as the total number of infections is approximately three [95% prediction interval: 2–8] times higher than the number of confirmed cases.

Considering the urgent need for population-based seroprevalence studies in order to measure the actual progress of the COVID-19 pandemic, it is also critically important to assess whether local measurements could be representative of the corresponding total population. Analysing recent local seroprevalence estimates for the USA, Italy and Germany suggests that they are likely not representative of the total population, in particular in Germany. Local seroprevalence estimates may be biased due to false test results and to population samples that are not nationally representative.^29^

Our model estimates build on two key assumptions, that at best, only partially hold. Our first key assumption implies that COVID-19 deaths are fairly accurately recorded. However, COVID-19 deaths may be misreported, particularly in regions that are heavily affected by the pandemic.^30^ Reporting delays and inconsistent practices for defining and testing COVID-19 deaths may also influence the accuracy of reported deaths.^12^ If the numbers of reported deaths were too small, the infection estimates would be biased downward, and vice versa. However, if the amount of reporting bias is known, for example through studies gauging COVID-19-related excess mortality,^18–20^ our approach could easily incorporate this information.

Our second key assumption implies that infection fatality rates from a reference country are (i) fairly accurately recorded and (ii) become applicable in a target population through proper scaling based on remaining life expectancy. Infection fatality rates from a reference country could be biased due to, for example, test errors that can lead to misclassification of deaths. Watson and Brush^31^ note that COVID-19 tests have high specificity and only moderate sensitivity. Kumleben *et al*.^32^ point out that there can be many false-positives despite high test specificity if many tests are conducted and infection prevalence is low. This could result in over-reporting COVID-19 deaths. However, recent results on excess mortality^18–20^ indicate that deaths are more likely to be under-reported. Another source of bias is misspecification in the statistical model used to estimate reference infection fatality rates. In the case of Hubei, this should be only a minor concern as Verity *et al*.^21^ have run several robustness checks. Nevertheless, Supplementary Appendix 5, available as Supplementary data at *IJE* online, shows how infection estimates increase when they are based on scaling French (instead of Chinese) infection fatality rates.^33^ If data are (or become) available, we recommend use of infection fatality rates of population-representative serological studies, as it would avoid circling effects between modelling approaches.

Although scaling the infection fatality rates between a reference and a target country increases the applicability of our estimation approach, such borrowing and scaling strategies cannot fully reflect country-specific trends. We argue that remaining lifetime is a useful marker to account for overall cross-country differences in age structure, health conditions and medical services, but also acknowledge that it cannot directly account for cross-country differences in, for example, the progress of the pandemic, the control measures taken and their acceptance in each population in order to prevent medical services from becoming overburdened. Scaling IFRs could be substantially impaired if reference and target population considerably differ: (i) in structure and distribution of major diseases that affect both vulnerability to COVID-19 and remaining lifetime; and (2) in the occupancy rate of medical services caused by different levels of preparedness for dealing with this pandemic.

Considering the rapidly changing pandemic, it is important to note that the proposed model can account for time-varying input parameters. This is useful, as not only the numbers of deaths change on a daily basis, but also IFRs may decrease as experience with best treatment practices accumulates.^34^^,^^35^

Our model can account for the duration between disease onset and death, which may be several weeks,^26–28^ without the need to change any equations. Supplementary Appendix 6, available as Supplementary data at *IJE* online, compares estimated infections as of 23 July 2020, with confirmed cases 18 days ago, which results in an increase of unknown infections. Not adjusting for this time lag leads to infection estimates that are generally too low. More specifically, this underestimation is likely to be greater or smaller when infection numbers increase or decrease. However, data about time to death are uncertain and vary by source.^26^^,^^27^

Our demographic scaling model estimates COVID-19 infections in a simple and fast manner in settings with rich and poor data. It can be the only option in situations in which the detailed data needed for precise estimation are unavailable and population-representative seroprevalence studies are lacking. Our model can be implemented broadly and provides useful information about the magnitude of the unknown number and prevalence of infections in countries worldwide. It is also a suitable tool to quantify the deviation of local seroprevalence estimates from their corresponding national mean. The model outcomes can be used in decision making and as input in more advanced models.^1^^,^^6^^,^^7^^,^^36–38^ Moreover, as the information about the key input parameters of our approach—deaths and infection fatality rates—improves, it will produce increasingly accurate infection estimates.

## Supplementary data

Supplementary data are available at *IJE* online.

## Supplementary Material

dyaa198_Supplementary_Data
