# Supplementary material for: A demographic scaling model for estimating the total number of COVID-19 infections
Source: Int J Epidemiol. 2020 Dec 8;49(6):1963–71. doi: 10.1093/ije/dyaa198 (PMC7799106; doi:10.1093/ije/dyaa198)
Supplement: dyaa198_Supplementary_Data [file dyaa198_supplementary_data.pdf]

Supplementary material

A demographic scaling model for estimating the total number of COVID-19 infections

Christina Bohk-Ewald, Christian Dudel, Mikko Myrskylä

Supplementary Appendix 1

COVID-19 infection fatality rates

To estimate COVID-19 infections with the introduced demographic scaling model for the 10 countries with most COVID-19 deaths as of July 23, 2020, we borrow and scale the infection fatality rates of Hubei, China, as published by Verity and colleagues.[1] For the sake of convenience and transparency, we list these infection fatality rates (the mode as well as the lower and upper bound of the 95% credible interval) in Supplementary Table S1.

Supplementary Table S1: Infection fatality rates for Hubei, China

	**Infection fatality rates, Hubei, China**		
**Age group**	**Mode**	**Lower 95%**	**Upper 95%**
0-9	0.000016	0.00000185	0.000249
10-19	0.00007	0.000015	0.0005
20-29	0.00031	0.00014	0.00092
30-39	0.00084	0.00041	0.00185
40-49	0.0016	0.00076	0.0032
50-59	0.006	0.0034	0.013
60-69	0.019	0.011	0.039
70-79	0.043	0.025	0.084
80+	0.078	0.038	0.133

Infection fatality rates (mode and lower and upper bound of 95% credible interval) by 10-year age groups for Hubei, China. Data source: Verity and colleagues.[1]

Supplementary Appendix 2

Estimate COVID-19 infections based on scaling infection fatality rates between two countries via thanatological age

It takes four steps to estimate COVID-19 infections for a country of interest with the introduced demographic scaling model, which maps, e.g., the infection fatality rates of Hubei, China, onto a country of interest via the thanatological age.

1. Ungroup the reference country's infection fatality rates IFR_x from 10-year age groups into single years of age using a cubic smoothing spline via the R-function *smooth.spline*.

2. Ungroup the remaining life years (e_x), taken from abridged life tables of the United Nations World Population Prospects,[2] for both the reference country and the country of interest, so that they are on a more detailed fine grid: age measured in years using two decimal places (i.e., 0, 0.01, 0.02, …, 99.99, 100) and remaining lifetime measured in years using three decimal places. Note that the resolution of this grid can be adjusted in the R source code provided at https://github.com/christina-bohk-ewald/demographic-scaling-model.

3. Map the ungrouped infection fatality rates (from step 1) of the reference country onto the country of interest via thanatological age (i.e., interpolated remaining lifetime from step 2) in order to better match the context with respect to age structure, preconditions, and medical services. The scaled infection fatality rates for the 10 countries with the most COVID-19 deaths as of July 23, 2020, are shown in Supplementary Figure S1.

4. Calculate the number of COVID-19 infections (I) based on equation $I = \sum_x D_x \,/IFR_x$.

Supplementary Figure S1 displays the scaled IFR_x for the 10 countries with the most deaths attributable to COVID-19 as of July 23, 2020. Supplementary Tables S2 and S3 list the

corresponding IFR_x by 10-year age groups, as well as the crude IFR_x for each of those countries, based on central estimates and the lower bound of the 95% prediction interval.

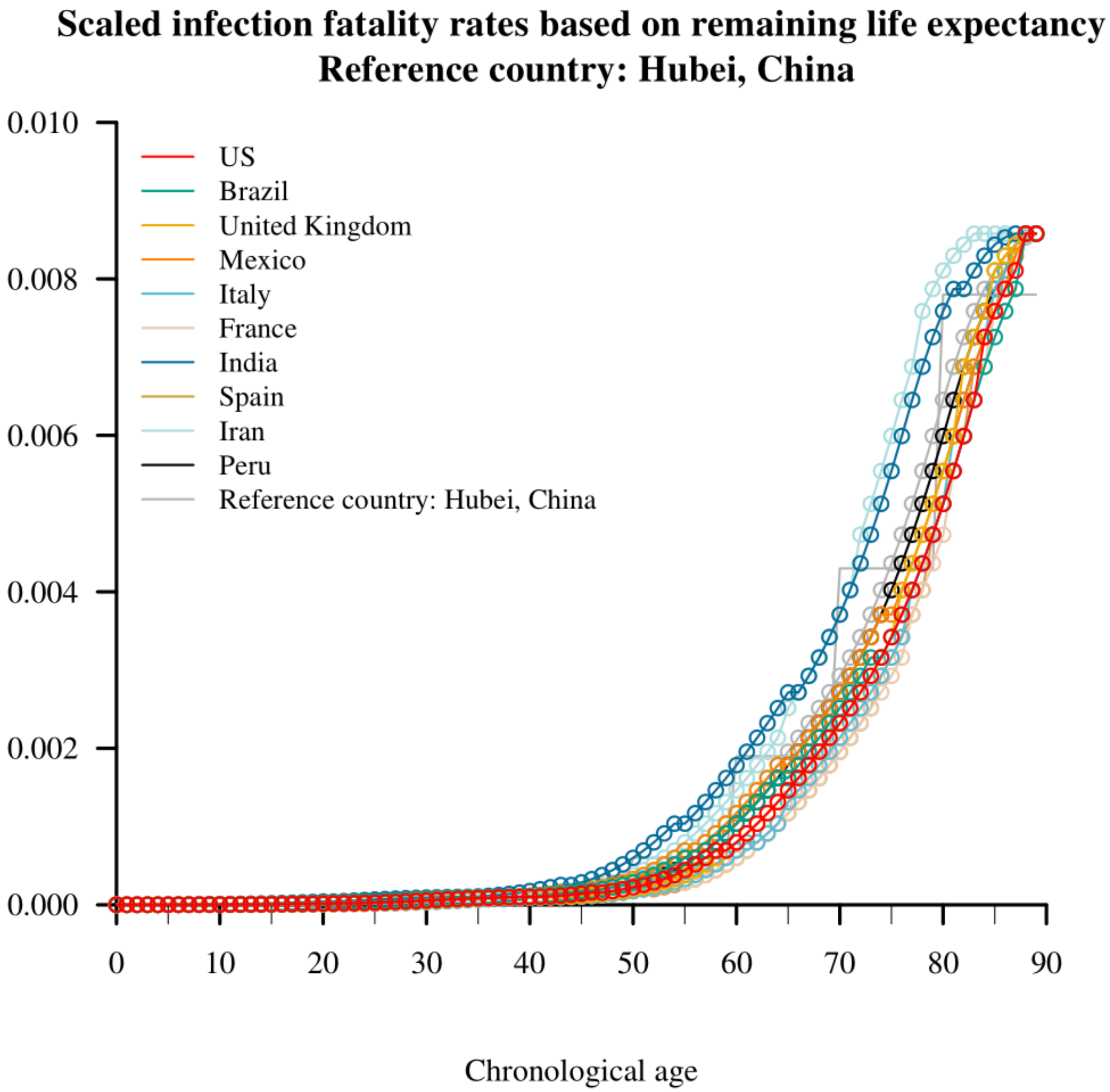

Supplementary Figure S1. Scaled infection fatality rates based on remaining lifetime of China Shown are the 10 countries that have the largest numbers of reported deaths from COVID-19 as of July 23, 2020. Own calculations using data from Verity and colleagues[1] and abridged life tables of United Nations World Population Prospects.[2]

Supplementary Table S2: Scaled central estimates of infection fatality rates (IFR)

	Age-specific Infection Fatality Rates									Crude IFR
	**0-9**	**10-19**	**20-29**	**30-39**	**40-49**	**50-59**	**60-69**	**70-79**	**80+**	**All ages**
**US**	0.000016	0.000070	0.00028	0.00079	0.00138	0.00442	0.01420	0.03389	0.07110	0.02721
**Brazil**	0.000021	0.000094	0.00040	0.00088	0.00167	0.00556	0.01643	0.03473	0.06987	0.03044
**UK**	0.000011	0.000051	0.00023	0.00074	0.00122	0.00393	0.01420	0.03531	0.07526	0.02593
**Mexico**	0.000024	0.000109	0.00042	0.00092	0.00183	0.00641	0.01807	0.03789	0.07304	0.03295
**Italy**	0.000010	0.000037	0.00017	0.00063	0.00113	0.00341	0.01238	0.03232	0.07328	0.02266
**France**	0.000010	0.000038	0.00017	0.00063	0.00113	0.00310	0.01140	0.02983	0.07051	0.02178
**India**	0.000043	0.000200	0.00064	0.00113	0.00306	0.01065	0.02565	0.05408	0.08244	0.04473
**Spain**	0.000010	0.000033	0.00017	0.00063	0.00113	0.00301	0.01140	0.03090	0.07195	0.02158
**Iran**	0.000021	0.000092	0.00036	0.00091	0.00200	0.00787	0.02370	0.05792	0.08489	0.03859
**Peru**	0.000018	0.000081	0.00036	0.00086	0.00160	0.00525	0.01732	0.03972	0.07574	0.03129

Shown are the 10 countries that have the largest numbers of reported deaths from COVID-19 as of July 23, 2020. Own calculations based on the age-specific modal infection fatality rates of Verity and colleagues[1] and abridged life tables of the United Nations World Population Prospects.[2]

Supplementary Table S3: Scaled lower 95% estimates of infection fatality rates (IFR)

	Age-specific Infection Fatality Rates									Crude IFR
	**0-9**	**10-19**	**20-29**	**30-39**	**40-49**	**50-59**	**60-69**	**70-79**	**80+**	**All ages**
**US**	0.000002	0.000015	0.000120	0.00039	0.00062	0.00251	0.00801	0.02040	0.03568	0.01253
**Brazil**	0.000002	0.000025	0.000192	0.00042	0.00080	0.00317	0.00932	0.02099	0.03527	0.01463
**UK**	0.000002	0.000008	0.000096	0.00037	0.00054	0.00222	0.00801	0.02102	0.03708	0.01098
**Mexico**	0.000002	0.000032	0.000205	0.00043	0.00091	0.00363	0.01036	0.02268	0.03635	0.01593
**Italy**	0.000002	0.000004	0.000064	0.00032	0.00050	0.00191	0.00696	0.01938	0.03641	0.00849
**France**	0.000002	0.000004	0.000064	0.00032	0.00050	0.00172	0.00638	0.01792	0.03546	0.00824
**India**	0.000006	0.000079	0.000322	0.00050	0.00170	0.00595	0.01543	0.02959	0.03947	0.02226
**Spain**	0.000002	0.000003	0.000064	0.00032	0.00050	0.00166	0.00638	0.01854	0.03596	0.00743
**Iran**	0.000002	0.000024	0.000168	0.00043	0.00102	0.00443	0.01413	0.03095	0.04028	0.01728
**Peru**	0.000002	0.000020	0.000167	0.00042	0.00076	0.00298	0.00992	0.02345	0.03725	0.01449

Shown are the 10 countries that have the largest numbers of reported deaths from COVID-19 as of July 23, 2020. Own calculations based on the age-specific lower 95% infection fatality rates of Verity and colleagues[1] and abridged life tables of the United Nations World Population Prospects.[2]

Supplementary Appendix 3

Age pattern of COVID-19 deaths

Supplementary Figure S2 shows the pattern over 10-year age groups of COVID-19 deaths using data provided by Dudel and colleagues.[3] Based on all available death age profiles, age standardized and normalized to sum to one, we calculate the average pattern over age that we use to split total deaths.

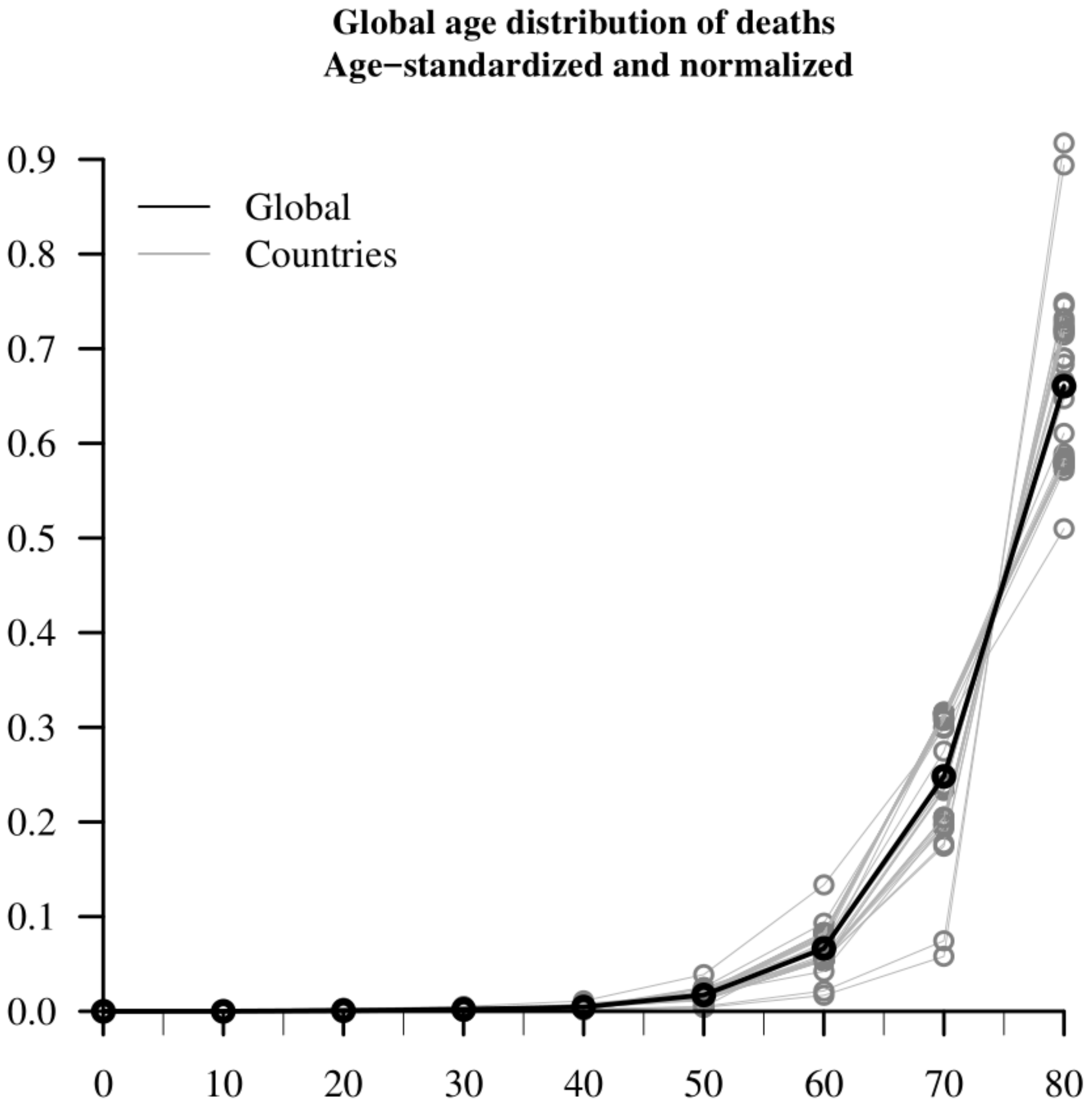

Supplementary Figure S2. Estimated global pattern over age of deaths attributable to COVID-19. Own calculations using data provided in Dudel and colleagues.[3]

Supplementary Appendix 4

Estimation of COVID-19 infections using the unadjusted model

Supplementary Figures S3 and S4 illustrate the estimated numbers and population shares of COVID-19 infections based on scaling Chinese modal infection fatality rates via chronological age in the unadjusted model, from January 22 to July 23, 2020.

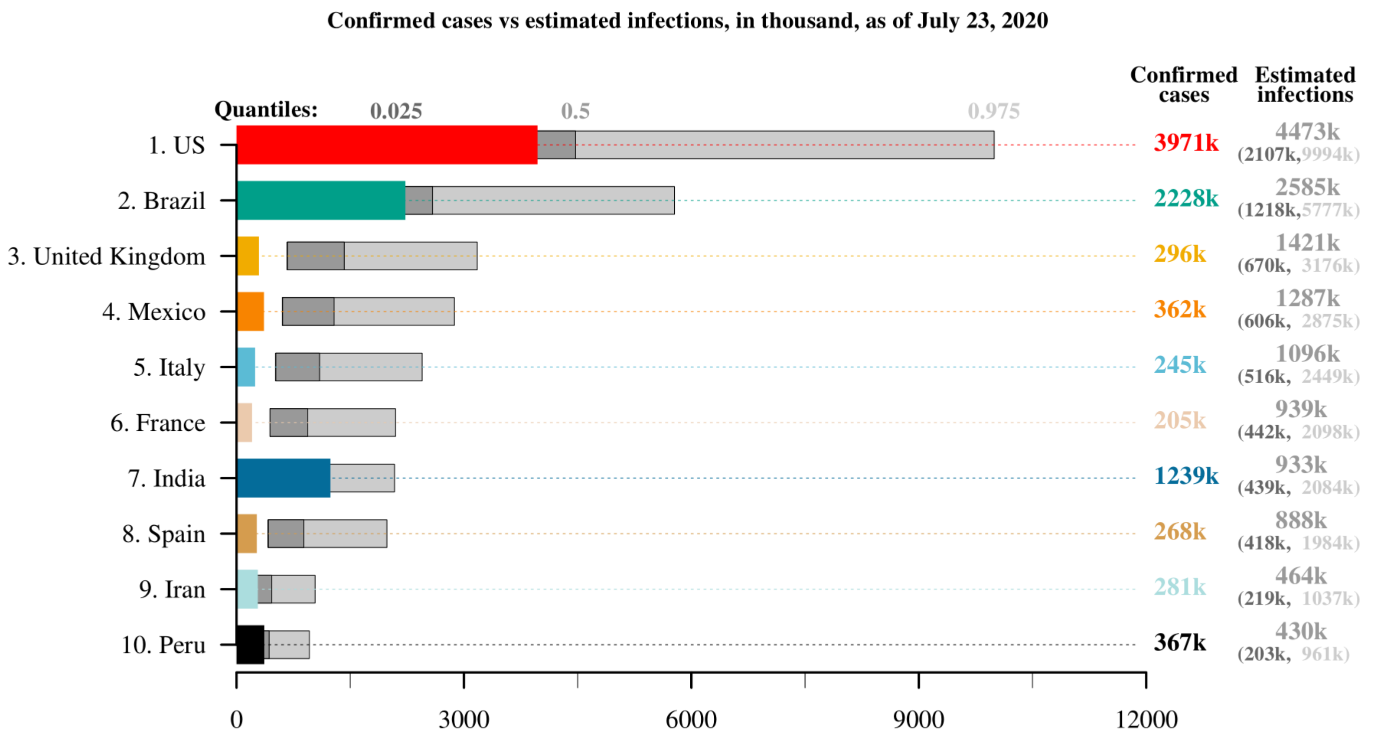

Supplementary Figure S3. Confirmed cases versus estimated infections (with scaling infection fatality rates based on chronological age) Confirmed cases (colored bars) and estimated COVID-19 infections (quantiles 0.025, 0.5, and 0.975; grey bars) based on unadjusted model. Shown are the 10 countries that have the largest numbers of reported deaths from COVID-19 as of July 23, 2020. Own calculations using data from Verity and colleagues,[1] United Nations World Population Prospects,[2] and Johns Hopkins University Center for Systems Science and Engineering.[4]

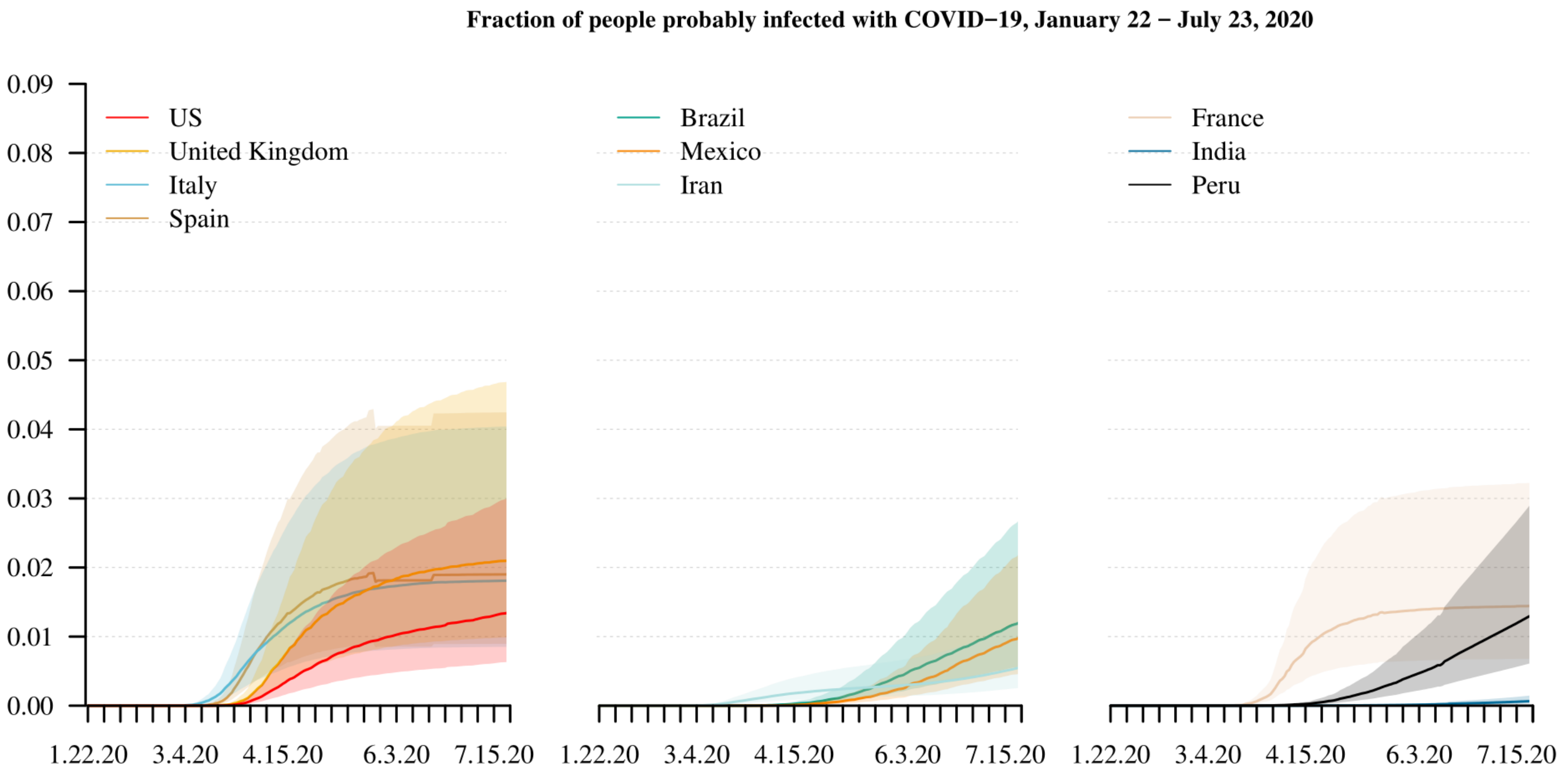

Supplementary Figure S4. Estimated COVID-19 infection prevalence (with scaling infection fatality rates based on chronological age) Estimated prevalence of COVID-19 infections (quantiles 0.025, 0.5, and 0.975), based on unadjusted model using, from January 22 to July 23, 2020. Shown are the 10 countries that have the largest numbers of reported deaths from COVID-19 as of July 23, 2020. Own calculations using data from Verity and colleagues,[1] United Nations World Population Prospects,[2] and Johns Hopkins University Center for Systems Science and Engineering.[4]

Supplementary Appendix 5

Estimates based on French infection fatality rates using the adjusted model

Supplementary Figures S5 and S6 illustrate the estimated total numbers and population shares of COVID-19 infections based on the adjusted model that scales French infection fatality rates as reported by Salje and colleagues[5] via remaining life expectancy (thanatological age), from January 22 to July 23, 2020.

Across the 10 countries with the most COVID-19 deaths as of July 23, 2020, our central estimates suggest that the total number of infections is almost five times higher than the number of confirmed cases. However, the level of uncertainty is high, as the lower bound of the 95% credible interval suggests that, on average, there are almost three times as many infections as confirmed cases, and the upper bound indicates that there are close to 12 times as many infections as confirmed cases. Country-specific variation is high. Our central estimates for Italy suggest that the total number of infections is approximately 2.3 million, or slightly more than nine times higher than the number of country-specific confirmed cases. For the U.S., our modal estimate seven million infections is almost two times higher than the number of confirmed cases, and the upper bound of almost 23.3 million infections is almost six times higher than the number of confirmed cases. For India, where testing has been comparatively limited, we estimate that the total number of infections is smaller than the number of confirmed cases.

Based on the central estimates, as of July 23, 2020, we find that the prevalence of COVID-19 infections ranges from roughly 4% in Spain; to between 3.2% and 3.8% in Italy, the U.K., and France; to approximately 2.1% in the U.S.; to between 1.2% and 1.8% in Peru, Brazil, and Mexico; and to 0.6% or less in Iran and India. The uncertainty bounds are wide and range on average between 1.4% and 5.8%. The COVID-19 prevalence estimates are largest in Spain, Italy, and the U.K. with 10.6%, 9.9%, and 9.7%, respectively.

We find that the total numbers and prevalence of infections are estimated to differ between the two sets of infection fatality rates as reported by Verity and colleagues[1] and Salje and colleagues[5]. The infection estimates appear to be larger based on French infection fatality rates reported by Salje and colleagues.[5] Supplementary Figure S7 compares the two sets of infection fatality rates. It shows that the French infection fatality rates of Salje and colleagues[5] are lower for age groups 50-59, 60-69, and 70-79 and higher for age group 80+. Both sets of infection fatality rates are produced with a Bayesian model. They can be biased due to, e.g., model misspecification and misreported input data.

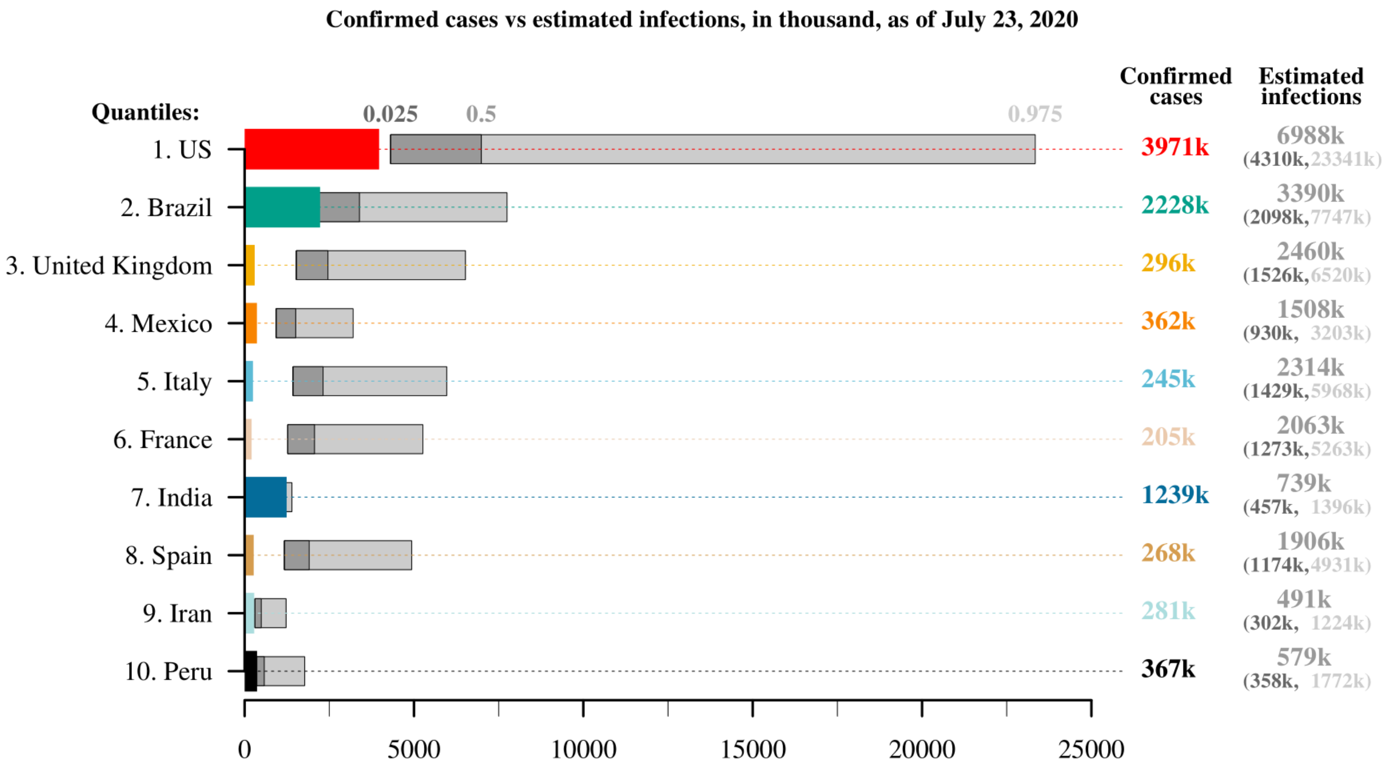

Supplementary Figure S5. Confirmed cases versus estimated infections (with scaling French infection fatality rates) Confirmed cases (colored bars) and estimated COVID-19 infections (quantiles 0.025, 0.5, and 0.975; grey bars) based on adjusted model that scales French infection fatality rates from Salje and colleagues[5]. Shown are the 10 countries that have the largest numbers of reported deaths from COVID-19 as of July 23, 2020. Own calculations using data from Salje and colleagues,[5] United Nations World Population Prospects,[2] and Johns Hopkins University Center for Systems Science and Engineering.[4]

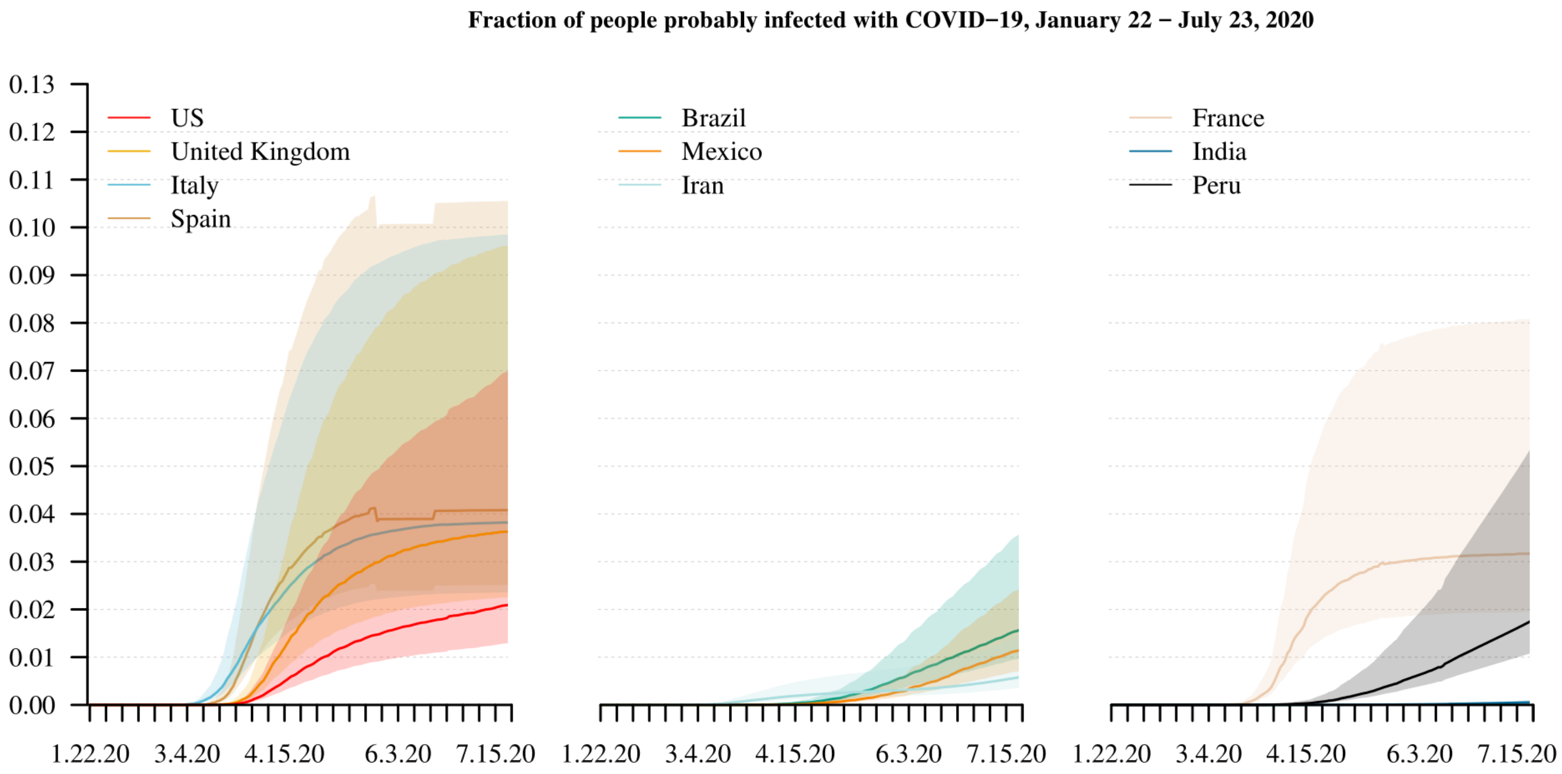

Supplementary Figure S6. Estimated COVID-19 infection prevalence (with scaling French infection fatality rates) Estimated population share of COVID-19 infections (quantiles 0.025, 0.5, and 0.975) based on adjusted model that scales French infection fatality rates from Salje and colleagues[5], from January 22 to July 23, 2020. Shown are the 10 countries that have the largest numbers of reported deaths from COVID-19 as of July 23, 2020. Own calculations using data from Salje and colleagues,[5] United Nations World Population Prospects,[2] and Johns Hopkins University Center for Systems Science and Engineering.[4]

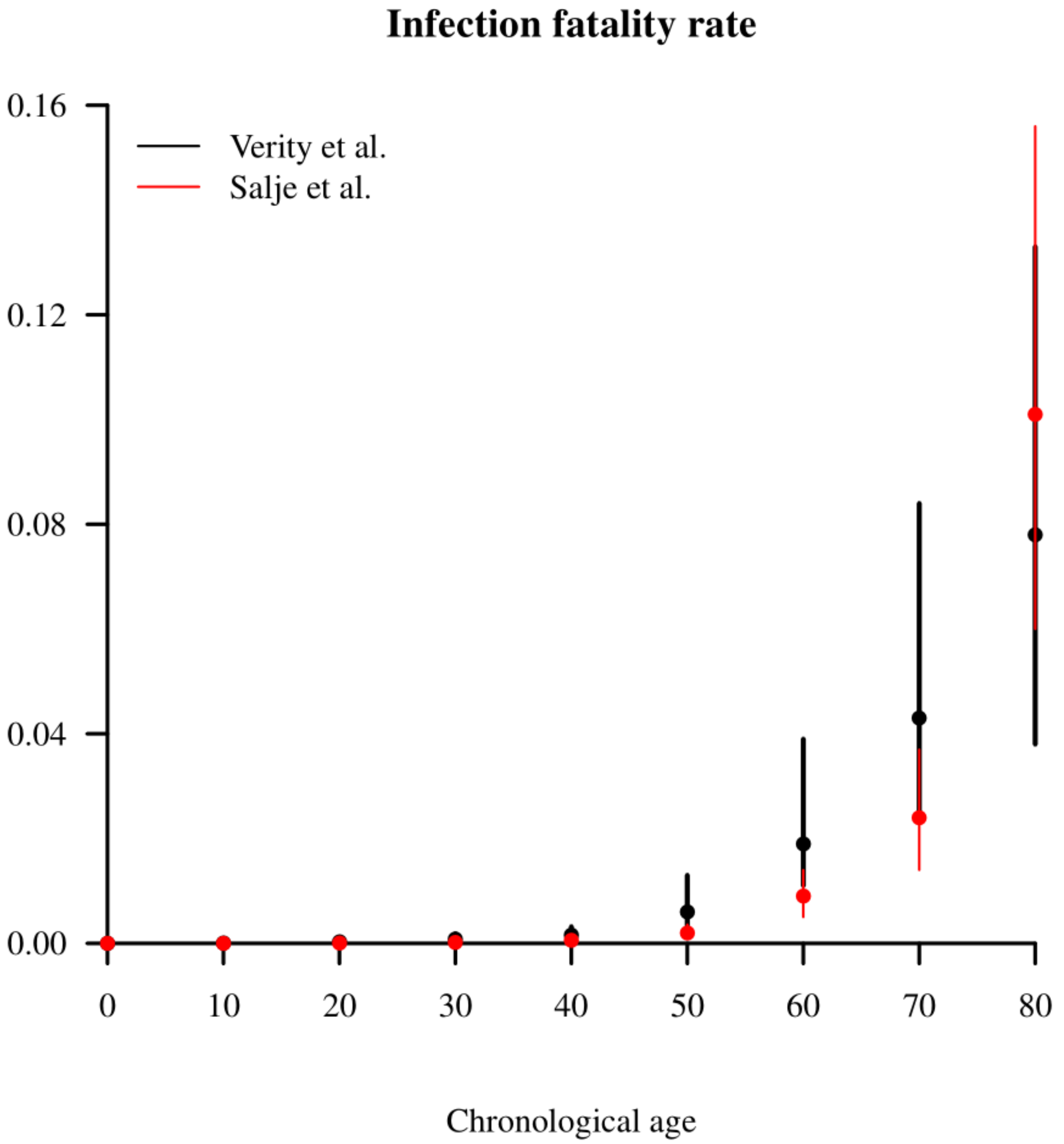

Supplementary Figure S7. Infection fatality rates of China and France Infection fatality rates (central estimate and 95% credible interval) reported by Verity and colleagues[1] (black) and by Salje and colleagues[5] (red) for 10-year age groups.

Supplementary Appendix 6

Account for time to death when estimating COVID-19 infections

Supplementary Figure S8 compares the estimated numbers of COVID-19 infections as of July 23, 2020, with the numbers of confirmed cases as of July 5, 2020, in order to account for the average time to death of 18.5 days, as reported in Zhou and colleagues.[6] We use the adjusted model and take as input infection fatality rates (IFR) of Verity and colleagues[1] scaled via remaining life expectancy (thanatological age) and latest accumulated deaths from COVID-19 as of July 23, 2020.

Across the 10 countries with the most COVID-19 deaths as of July 23, 2020, our central estimates suggest that the total number of infections is approximately four times higher than the number of confirmed cases. However, the level of uncertainty is high, as the lower bound of the 95% credible interval suggests that, on average, there are twice as many infections as confirmed cases, and the upper bound indicates that there are almost nine times as many infections as confirmed cases. Country-specific variation is high. Our modal estimates for Italy suggest that the total number of infections is approximately 1.6 million, or slightly more than six times higher than the number of country-specific confirmed cases. For the U.S., our modal estimate of 5.3 million infections is almost twice times higher than the number of confirmed cases, and the upper bound of 11.4 million infections is slightly more than four times higher than the number of confirmed cases. For India, where testing has been comparatively limited, we estimate that the total number of infections is very close to the number of confirmed cases.

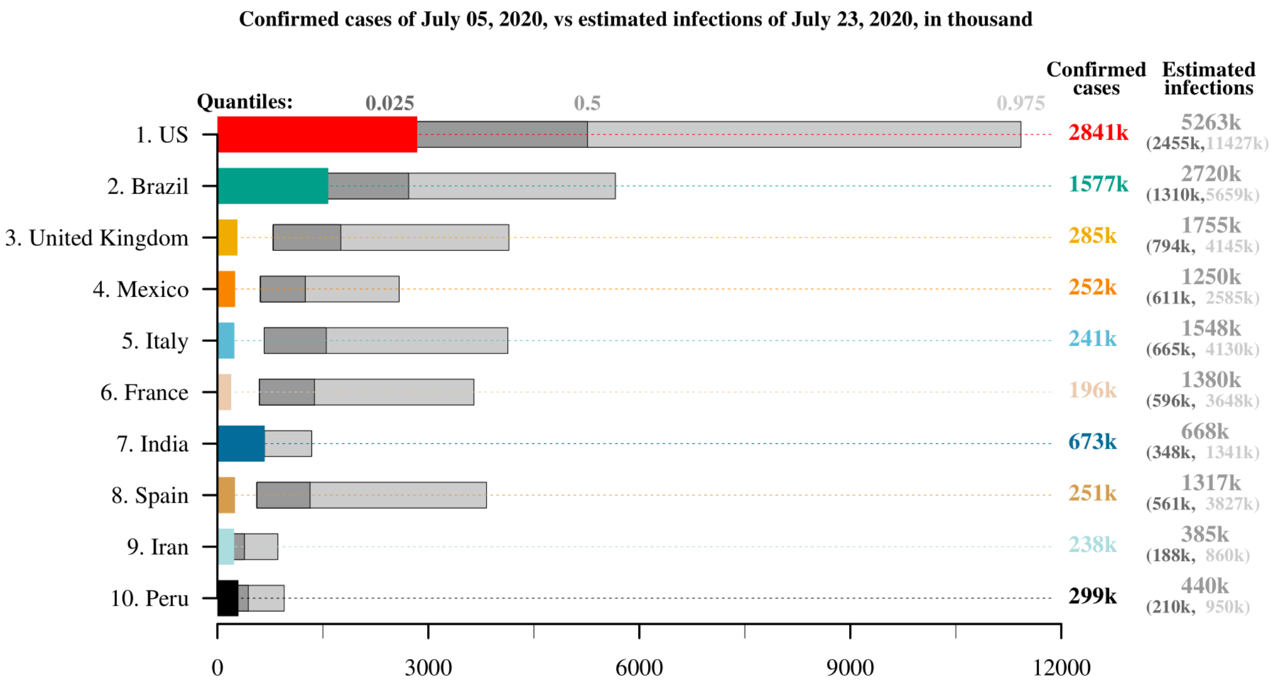

Supplementary Figure S8. Confirmed cases versus estimated infections (accounting for time to death) Confirmed cases (colored bars), as of July 5, 2020, and estimated total number of COVID-19 infections (quantiles 0.025, 0.5, and 0.975; grey bars), as of July 23, 2020, to account for average time to death: 18 days. Estimations are based on adjusted model that uses COVID-19 deaths as of July 23, 2020, and scaled Chinese infection fatality rates. Data are shown for the 10 countries that had the largest numbers of reported deaths from COVID-19 as of July 23, 2020. Own calculations using data from Verity and colleagues,[1] United Nations World Population Prospects,[2] and Johns Hopkins University Center for Systems Science and Engineering.[4]

Supplementary Appendix 7

Test coverage in ten countries with most reported COVID-19 deaths as of July 21, 2020

We show the positive association between the number of tests per million inhabitants and (i) the duration of the outbreak in Figure S9 and (ii) the cumulated number of confirmed cases per million inhabitants in Figure S10. Note that test coverage can increase in response to the extent of the outbreak and that higher levels of test coverage can, in turn, increase the chances to actually find cases.

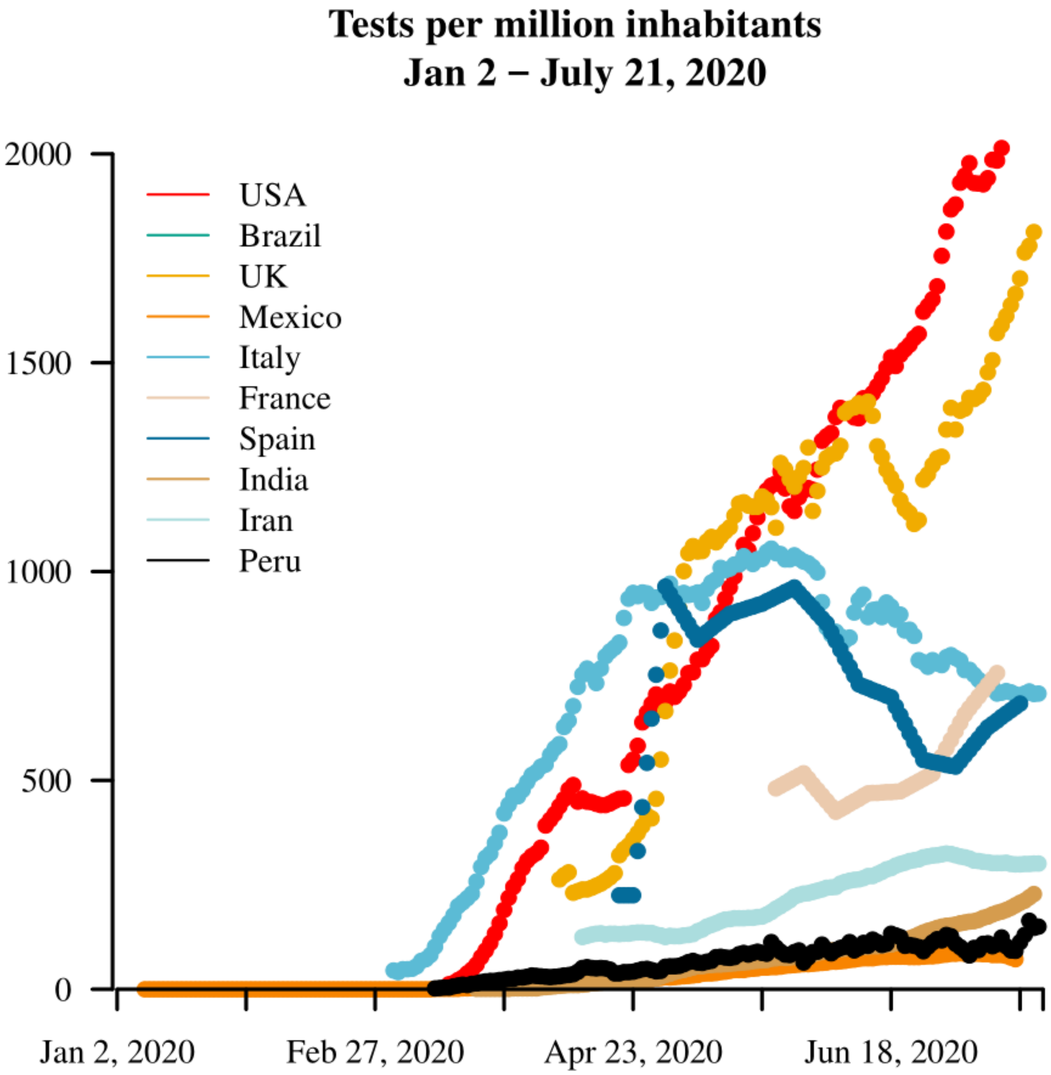

Supplementary Figure S9. Test coverage per million inhabitants over time Number of tests per million inhabitants (vertical axis) in the ten countries with most reported deaths as of July 21, 2020, from January 2, 2020, to July 21, 2020. Using data from www.ourworldindata.org and Johns Hopkins University Center for Systems Science and Engineering.[3]

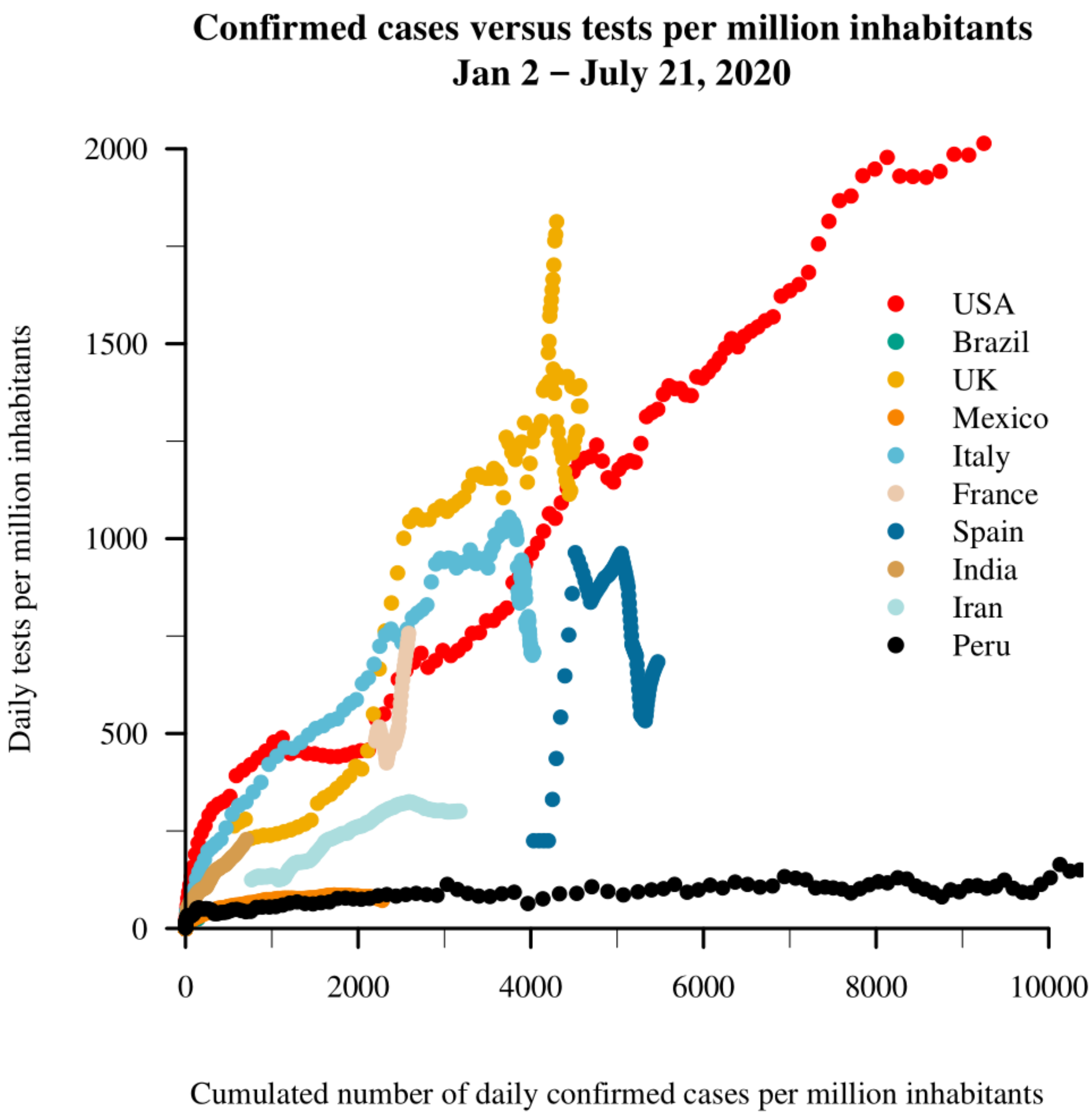

Supplementary Figure S10. Test coverage and cumulated number of confirmed cases per million inhabitants Number of tests per million inhabitants (vertical axis) versus cumulated number of confirmed cases (horizontal axis) in the ten countries with most reported deaths as of July 21, 2020, from January 2, 2020, to July 21, 2020. Using data from www.ourworldindata.org and Johns Hopkins University Center for Systems Science and Engineering.[3]

Supplementary Appendix References

1. Verity R, Okell LC, Dorigatti I, et al. Estimates of the severity of coronavirus disease 2019: a model-based analysis. *Lancet Infect Dis 2020;* published online March 30. DOI:10.1016/S1473-3099(20)30243-7.
2. United Nations, Department of Economic and Social Affairs, Population Division. (2019). Population Prospects 2019. Published online: https://population.un.org/wpp/. Download on April 17, 2020.
3. Dudel C, Riffe T, Myrskylä M, van Raalte A, Acosta E. Monitoring Trends and Differences in COVID-19 Case Fatality Rates Using Decomposition Methods: Contributions of Age Structure and Age-Specific Fatality. medRxiv, doi:10.1101/2020.03.31.20048397, 8 April 2020, preprint: not peer reviewed.
4. Johns Hopkins University CSSE. Novel Coronavirus (COVID-19) Cases Data. Published online: https://data.humdata.org/dataset/novel-coronavirus-2019-ncov-cases. Download on April 17, 2020.
5. Salje H, Tran Kiem C, Lefrancq N, et al. Estimating the burden of SARS-CoV-2 in France. *Science 2020;* **eabc3517**. DOI: 10.1126/science.abc3517.
6. Zhou F, Yu T, Du R, et al. Clinical course and risk factors for mortality of adult inpatients with COVID-19 in Wuhan, China: a retrospective cohort study. *The Lancet 2020*; **395(10229)**: 1054-1062. DOI:10.1016/S0140-6736(20)30566-3.
